# Supplementary material for: Cellular interfaces with hydrogen-bonded organic semiconductor hierarchical nanocrystals
Source: Nat Commun. 2017 Jul 21;8:91. doi: 10.1038/s41467-017-00135-0 (PMC5522432; doi:10.1038/s41467-017-00135-0)
Supplement: Supplementary file 1 — Supplementary Information [file 41467_2017_135_MOESM1_ESM.pdf]

File Name: Supplementary Information
Description: Supplementary Figures, Supplementary Table, Supplementary Note and Supplementary Methods.

File Name: Peer Review File
Description:

Supplementary Methods

Cell Culture. Hedgehog microcrystals were drop cast from chloroform onto glass slides coated with (3-Aminopropyl)triethoxysilane, APTES (Human embryonic kidney HEK cells) or a monolayer of n-octyltriethoxysilane, OTS (Rat basophilic leukemia RBL cells), which were used for cell seeding. These self-assembled monolayer modifications were done by first treating glass samples with oxygen plasma, then transfering them into a sealed glass chamber with an open vial of OTS or APTES, and then heating the chamber to 70˚C, 3h for APTES and 90˚C, 1h, for OTS. The samples were then rinsed with isopropanol and sonicated in isopropanol (APTES) or toluene (OTS) to remove physioadsorbed layers. Samples were then sterilized prior to cell culture with UV light. Human embryonic kidney 293 (HEK) cells were cultured in DMEM supplemented with L-glutamine (2 mM), streptomycin (100 µg/ml), penicillin (100 U/ml), and 10% fetal calf serum, the rat basophilic leukemia 2H3 (RBL) cells were grown in MEM supplemented with 10% fetal calf serum, 2 mM glutamine, 2 U/ml penicillin and 2 mg/ml streptomycin, and incubated at 37°C, 95% humidity and 5% CO_2.
Cell viability. CytoTox-Glo™ cytotoxicity assay from Promega was performed according to the producer protocol. Hedgehog crystals in $CHCl_3$ and QNC powder dispersed in EtOH were deposited directly into a 96-well flat-bottom polypropylene culture plate. The QNC thin film was evaporated on a PDMS-coated PET foil, cut, and adhered to the bottom of the wells. The total mass of QNC was kept the same for all experimental conditions. The plates were then UV sterilized and seeded with 2000 cells. The cytotoxicity was determined after 4, 24, 48 and 72h without culture media exchange over the course of the experiment.
Scanning electron microscopy of cell-nanocrystal interfaces. The samples of cell cultures on the QNC microcrystals were prepared as described above. After 4-12 h of culture, the cells were fixed in 2% glutaraldehyde solution in phosphate buffer saline for 1 hour at room temperature or overnight at 4 °C. The samples were then washed with PBS three times and post-fixed using 2% OsO_4 solution in PBS, and finally washed again five times with PBS. The samples were then dehydrated using an ethanol series of 30%, 50%, 60%, 70%, 80% (10 min), 90%, 95% and 3 × 100% (each step for 15 min) and then dried at room temperature. The samples were metalized with thin film of gold using metal evaporation or sputtering. The micrographs were obtained from scanning electron microscope Zeiss XB 1540 or JEOL JSM-7401F.

Supplementary Table 1. Summary of nanocrystal syntheses.

Shape	Ligand	Solvent	Duration	Size	Crystal phase
Hedgehog	Oleylamine	Chloroform	24 hours	3-4 µm with 50 nm needles	α_2
Hedgehog	Oleylamine	Oleylamine	24 hours	8-11 µm with 50 nm needles	α_2
Hedgehog	Oleylamine	DMF	24 hours	15-19 µm with 50 nm needles	γ
Agave	Butylamine	Chloroform	12 hours	20-25 µm	γ
Houseleek	Butylamine/DMAP	Chloroform	12 hours	18-22 µm	γ
Coral fungi	Methylamine	Ethanol/ chloroform	12 hours	20-25 µm	γ
Starflower	Butylamine/Cyclohexanone	Cyclohexanone	44 hours	5-6 µm	β
Chrysanthe-mum	5-Aminotetrazole	Chloroform	1 month	45-65 µm with 1µm wide and 50 nm thin leaves	β

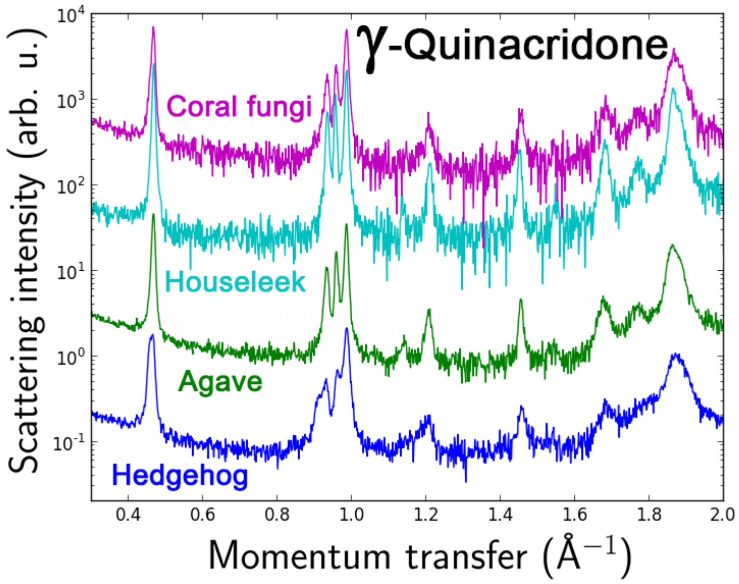

Supplementary Figure 1. X-ray diffraction of quinacridone nanoarchitectures. All quinacridone nanoarchitectures shown in Figure 1 of the main text, with different sizes and various bio-inspired shapes exhibit the same internal crystal structure, corresponding to the γ polymorph of quinacridone.

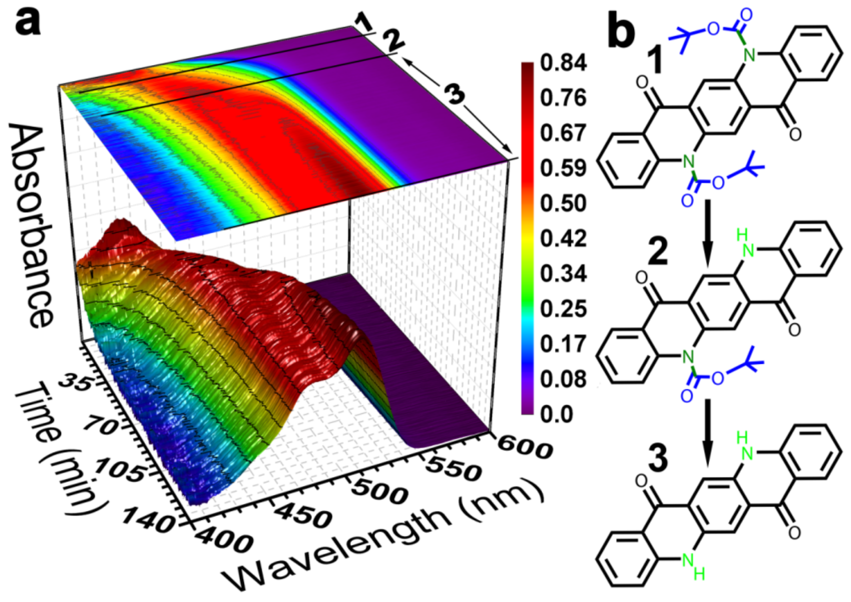

Supplementary Figure 2 | Optical tracking of the kinetics of the deprotection reaction. a, *In situ* absorbance spectra allow monitoring the dynamics of the deprotection reaction. **b**, the evolution can be sub-divided into 3 stages, corresponding to double-protected (**1**), mono-protected (**2**) and unprotected (**3**) quinacridone, constituting the monomer for crystal growth.

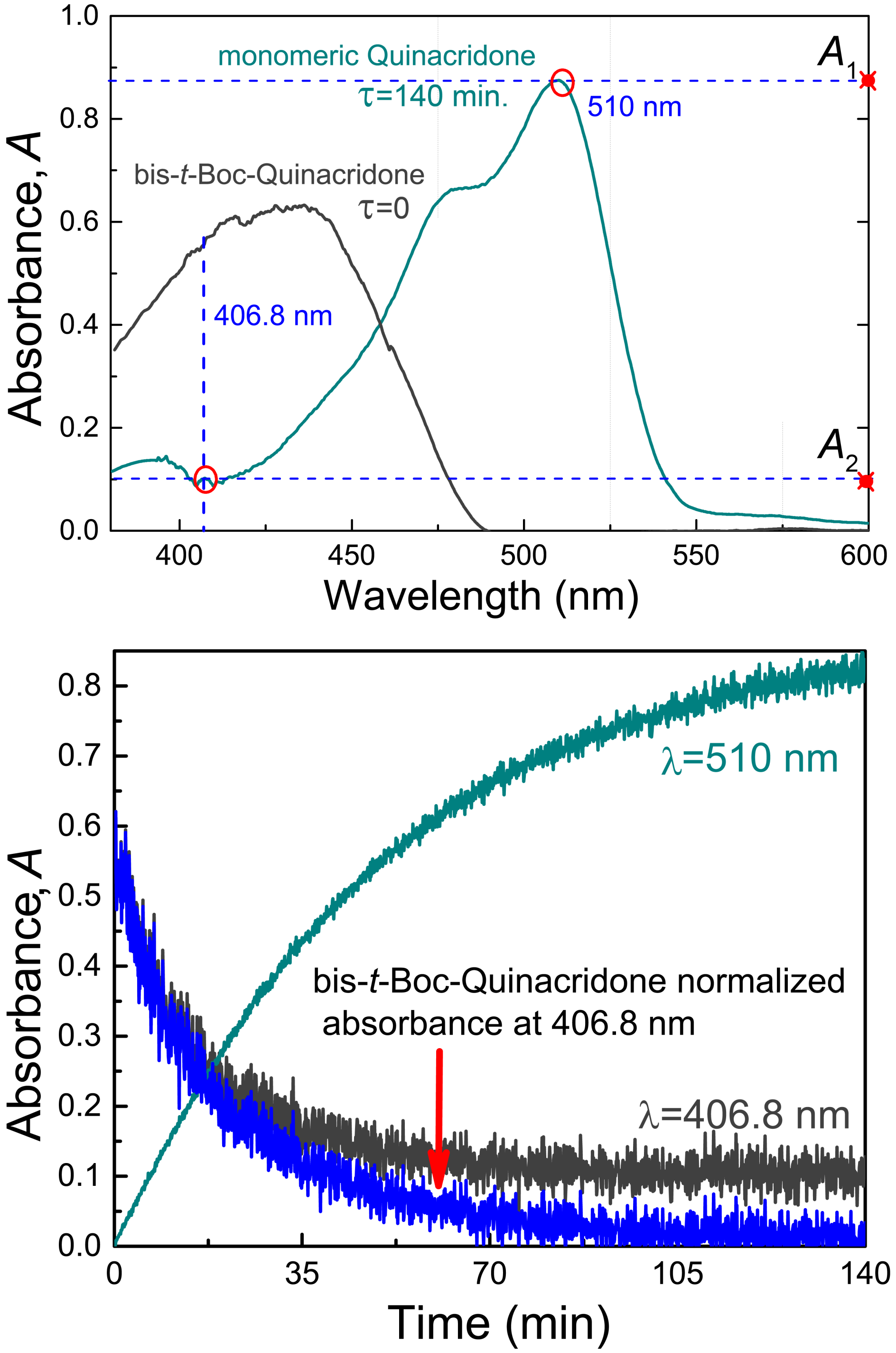

Supplementary Figure 3 | Time-dependant absorbance. a, Absorbance spectra at t=0 and t= 140 min. From the 140 min spectrum a weighting factor (A_1/A_2) is deduced to de-convolute the concentration transients of the bis-tBOC quinacridone and monomeric quinacridone at 406.8. **b**, Transient absorbance of monomeric quinacridone (510 nm), experimental transient absorbance at 406.8 nm, and de-convoluted transient absorbance due to bis-tBOC quinacridone.

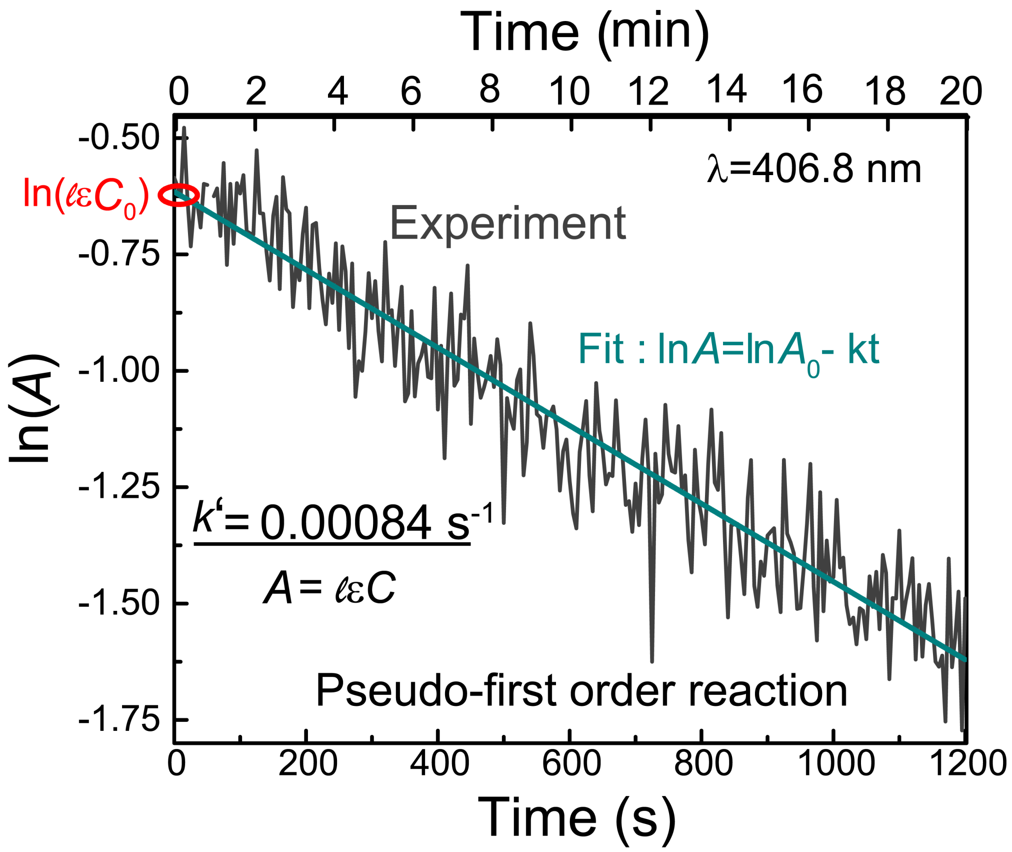

Supplementary Figure 4 | Dynamics of the bis-tBOC quinacridone deprotection reaction. The concentration of tBOC-QNC can be fitted by a mono-exponential decay, representing a pseudo-first order reaction. The absorbance (concentration) dependence of the tBOC-QNC exhibits a monoexponential decay, which can be fitted by $c=c_0e^{-kt}$, with a rate constant of $k=8.4*10^{-4}$ s^{-1}.

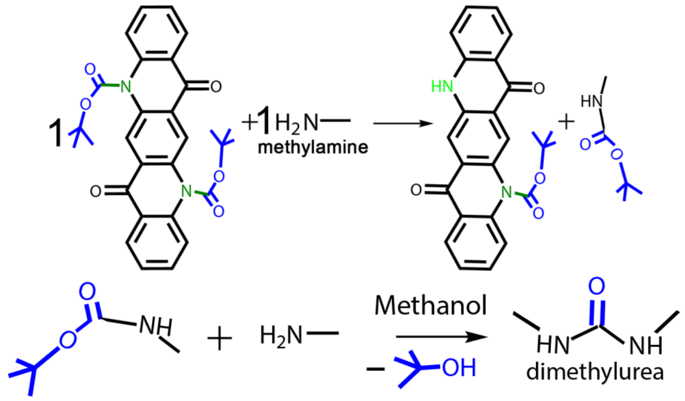

Supplementary Figure 5 | Reaction pathway of an excess of methylamine with bis-tBOC quinacridone, resulting in deprotection. If indeed the mechanism for deprotection is migration of the tBOC group to the nucleophillic methylamine, *t*-butyl methylcarbamate should be produced as a byproduct. The *t*-butyl methylcarbamate reacts further with methylamine to produce dimethylurea. Upon extracting the reaction mixture with methanol, white crystals are obtained from the methanol fraction and identified as dimethylurea using NMR (Supplementary Figure 6), thus allowing deduction of the above reaction mechanism.

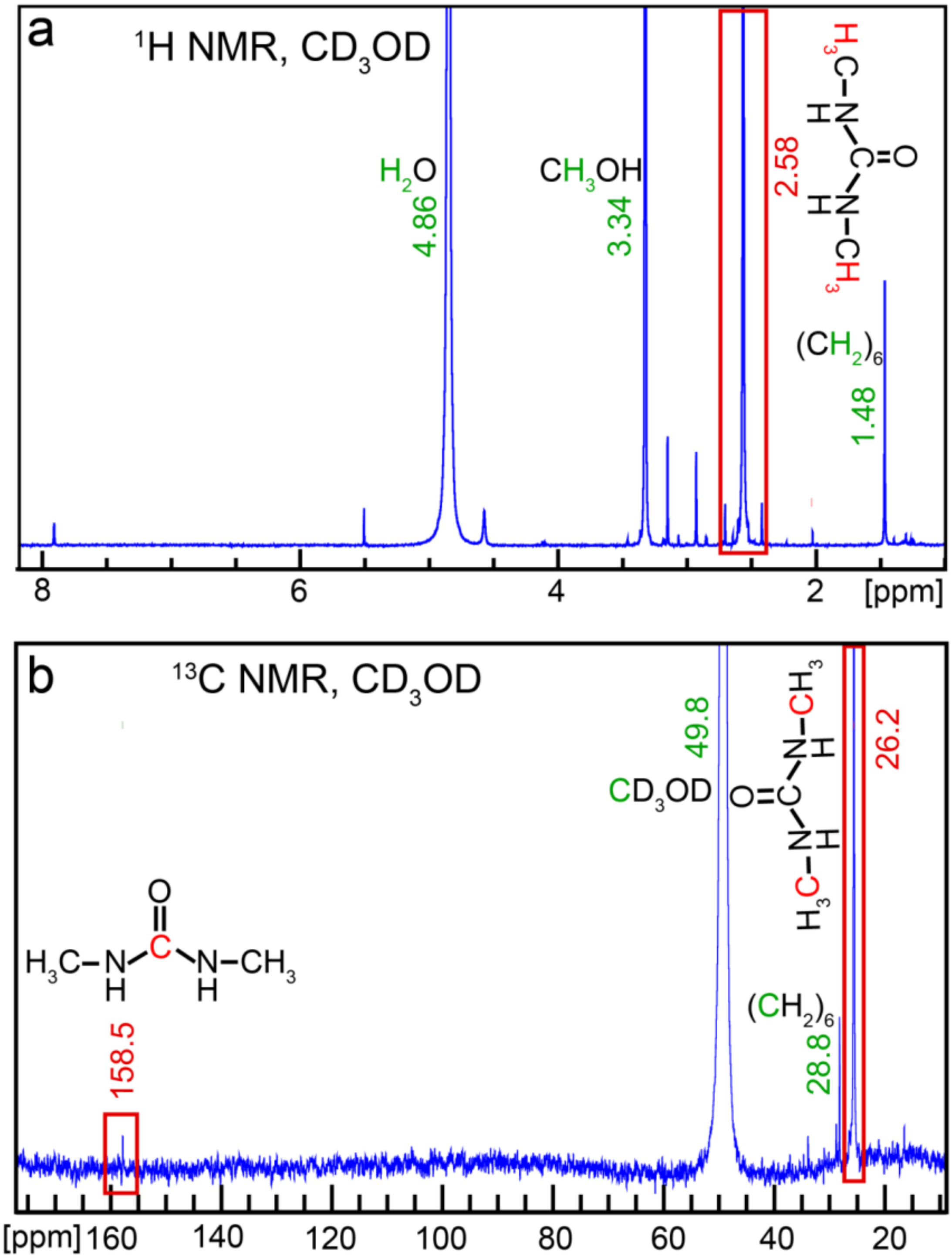

Supplementary Figure 6 | ^{1}H (a) and ^{13}C (b) NMR spectra, measured for the extracted side product, obtained after tBOC-QNC deprotection. The signal peaks marked with red boxes are originating from atoms marked with red colour in the structural formula. The peak positions are in agreement with literature data for dimethylurea. The presence of the latter confirms the chemical reaction suggested in Supplementary Figure 5.

Supplementary Note 1

Amine-induced deprotection mechanism. Experimentally it was found that the reaction dynamics changes with the length of the rest groups attached to the primary amines used to

react with tBOC-QNC. When short rest-groups are attached to the primary amine, the reaction is much faster, and the deprotection reaction can be finished after tens of minutes. The difference in reaction rate between long and short aliphatic amines reflects simply the different concentration of the $-NH_2$ moiety in the reaction solution (C_{RNH2}). Similarly, the reaction rate was observed to scale with the concentration of tBOC-QNC ($C_{tBOC\text{-}QNC}$). Thus, in principle the deprotection induced by the presence of primary amines represents a second order reaction (rate R), which is described the by the following rate equation:

$$\frac{dC}{dt} = R = kC_{tBOC-QNC} * C_{RNH_2} \qquad (1)$$

The experimentally observed monoexponential decay of the tBOC-QNC shown in Supplementary Figure 4, however, points to a pseudo-first order reaction. This pseudo-first order reaction regime is achieved in our experiments simply because the concentration of the primary amine (C_{RNH_2}) is several orders of magnitude higher than that of the tBOC-QNC ($C_{tBOC-QNC}$)[3]. In this case ($C_{RNH_2} >> C_{tBOC-QNC}$) the amine concentration can be considered to be hardly affected throughout the whole reaction time ($C_{RNH_2} = const.$) and the corresponding rate equation can be approximated by that of a first order reaction:

$$R = kC_{tBOC-QNC} * C_{RNH_2} = k'C_{tBOC-QNC} \qquad (2)$$

providing as solution the observed monoexponential decay of the tBOC-QNC concentration. Nevertheless, the reaction itself is of second order, otherwise the dependence of the reaction speed on the primary amine concentration could not be observed. We therefore conclude that the chemical reaction describing the deprotection reactions involves an equimolar interaction of the tBOC-QNC and the primary amine, as sketched in Supplementary Figure 5.

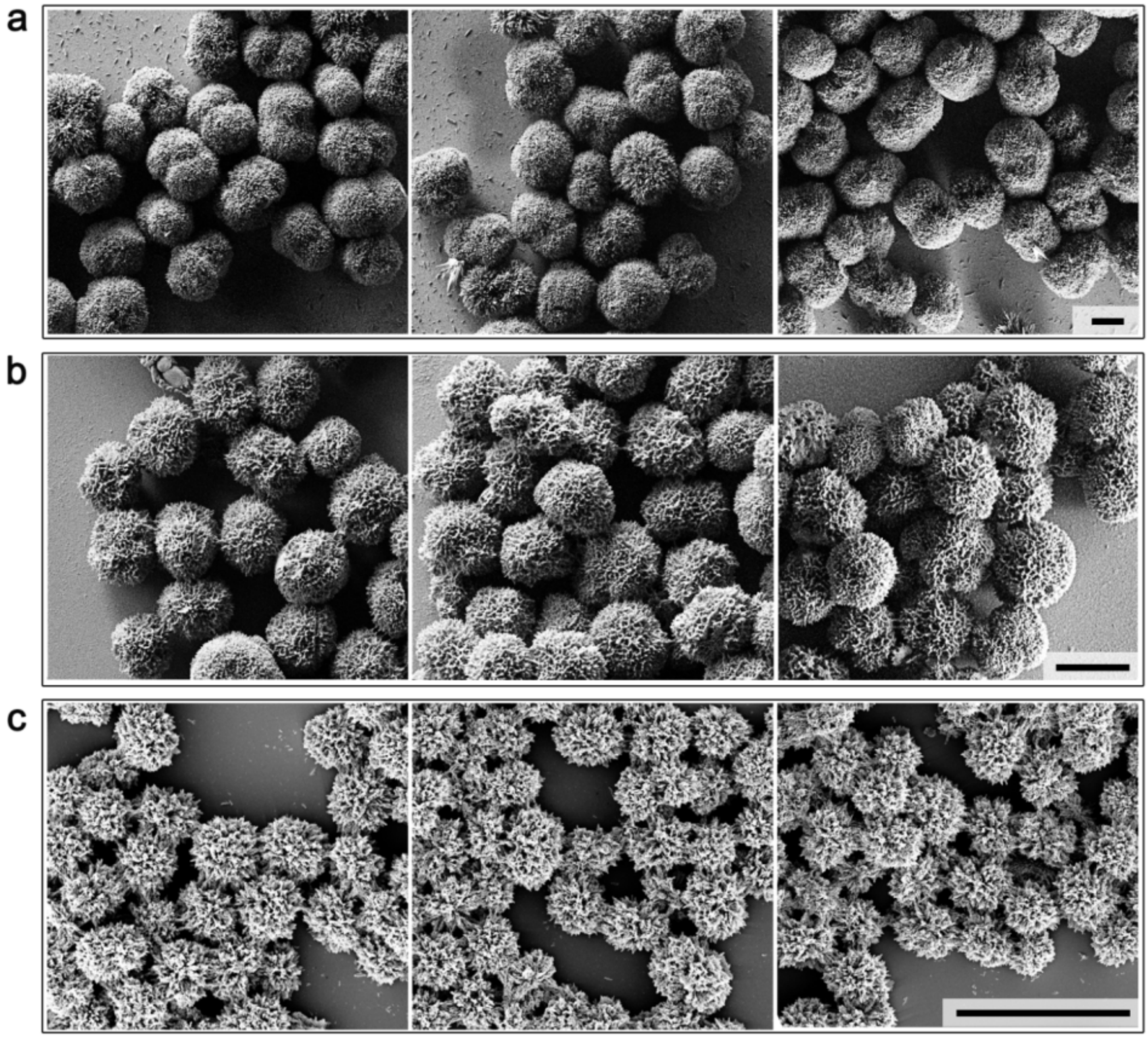

Supplementary Figure 7 | Hedgehog-shaped QNC nanoarchitectures. The electron micrographs evidence the spherical uniformity QNC nanoarchitectures with (a) 3.5, (b) 10, and (c), 18 μm diameters (10 μm scale bar in all cases). All particles were covered with 15 nm of Pt to avoid charging effects.

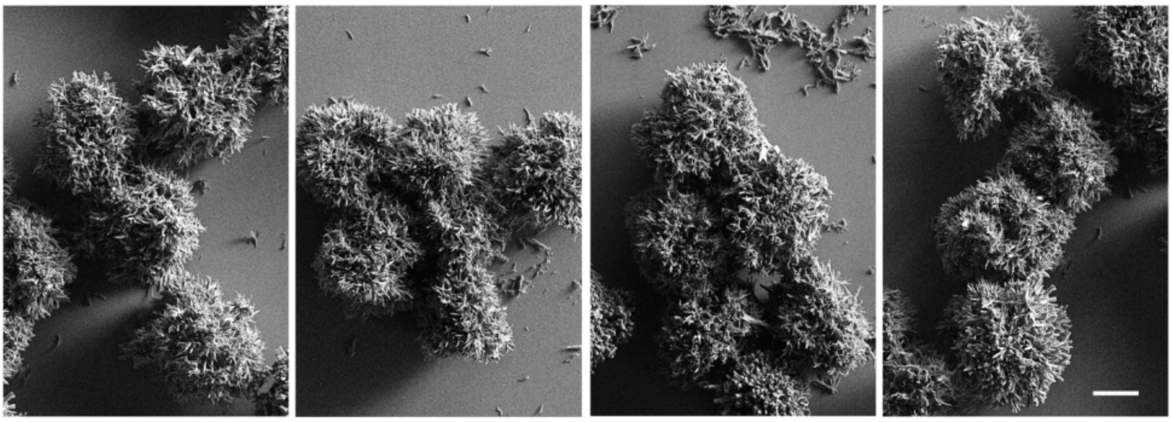

Supplementary Figure 8 | Coral fungi-shaped QNC nanoarchitectures. Electron micrographs evidencing the spherical outgrowth and branching of these nano/microarchitectures (10 μm scale bar).

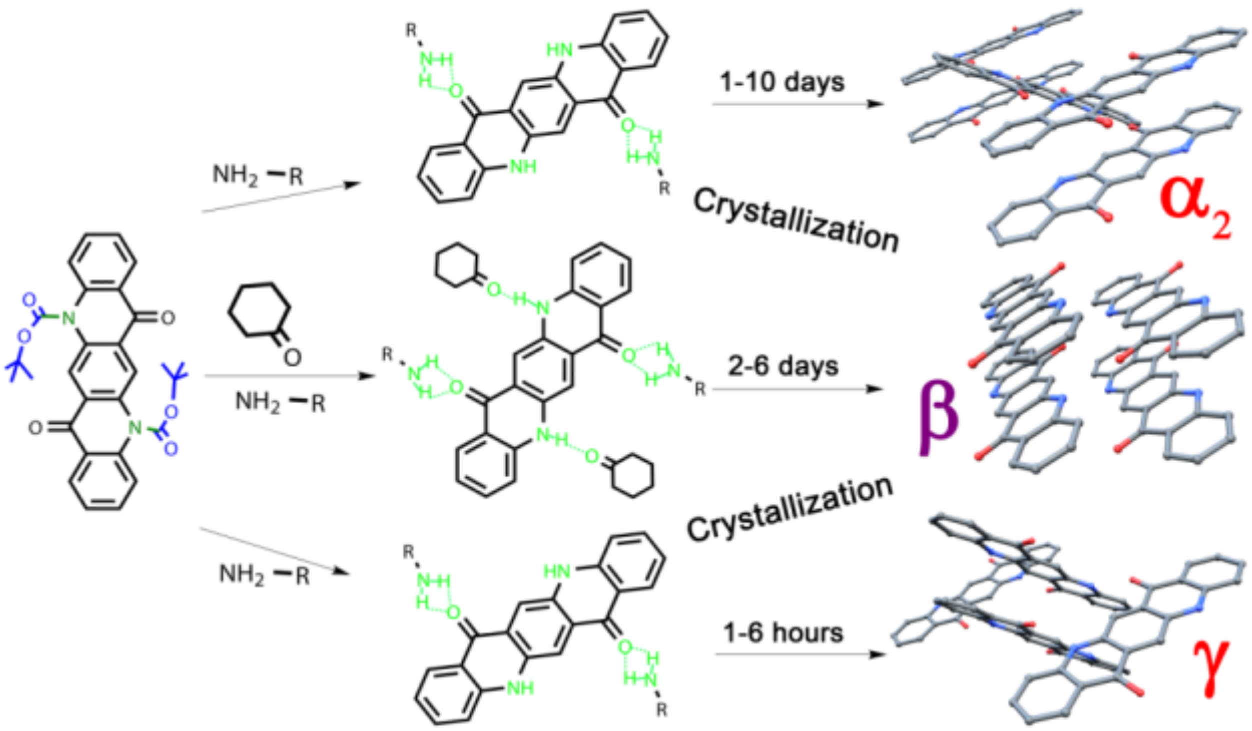

Supplementary Figure 9 | Three different crystallization paths for monomolecular QNC. Crystallization depends on the deprotection reaction rate and presence of coordinating solvent. Cyclohexanone influences the crystallization, for instance, by coordinating to NH groups which otherwise can interact readily with other QNC molecules.

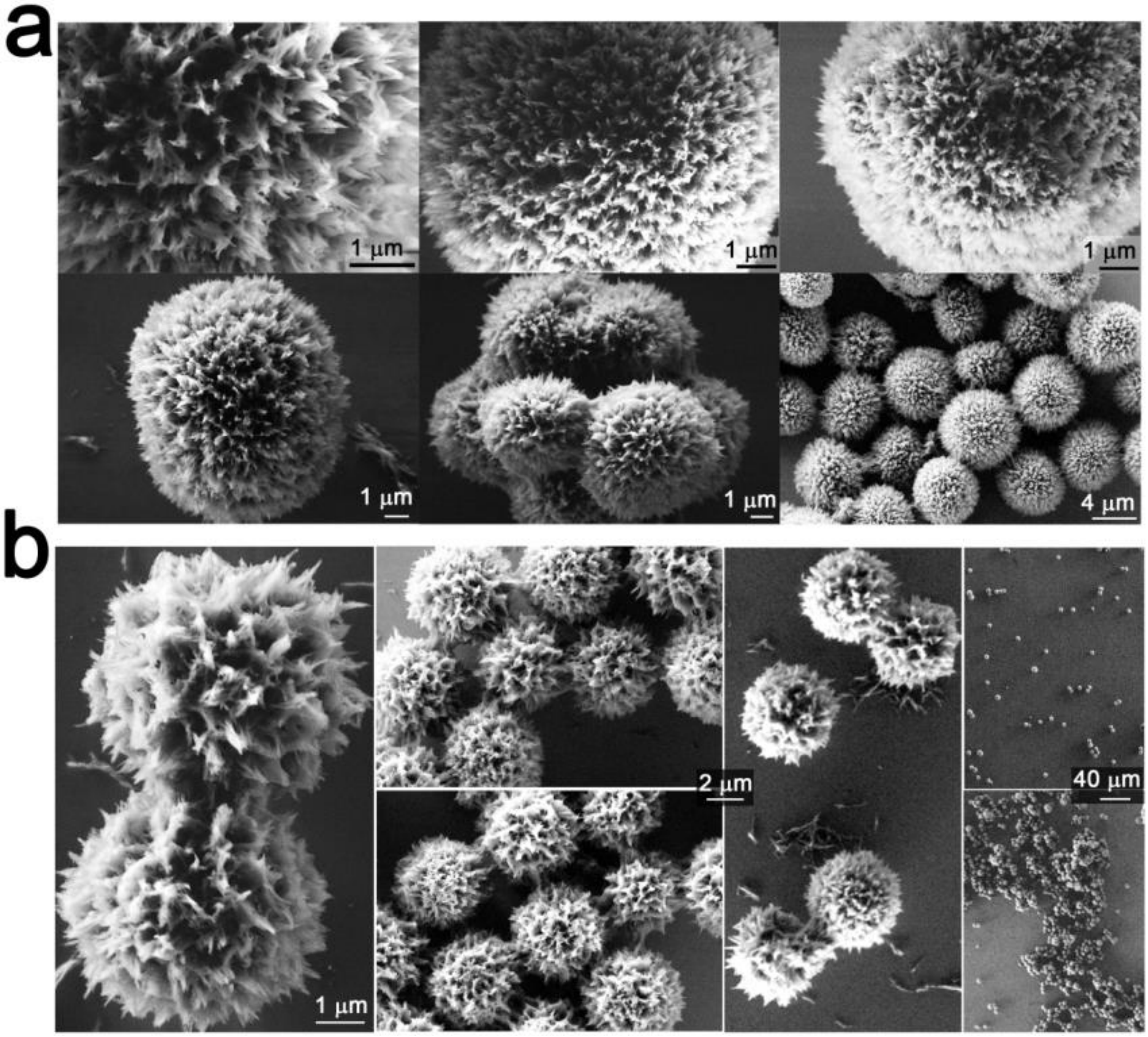

Supplementary Figure 10 | α_2 QNC hedgehogs. Different quinacridone superstructures which were grown into the α_2 polymorphic crystal phase.

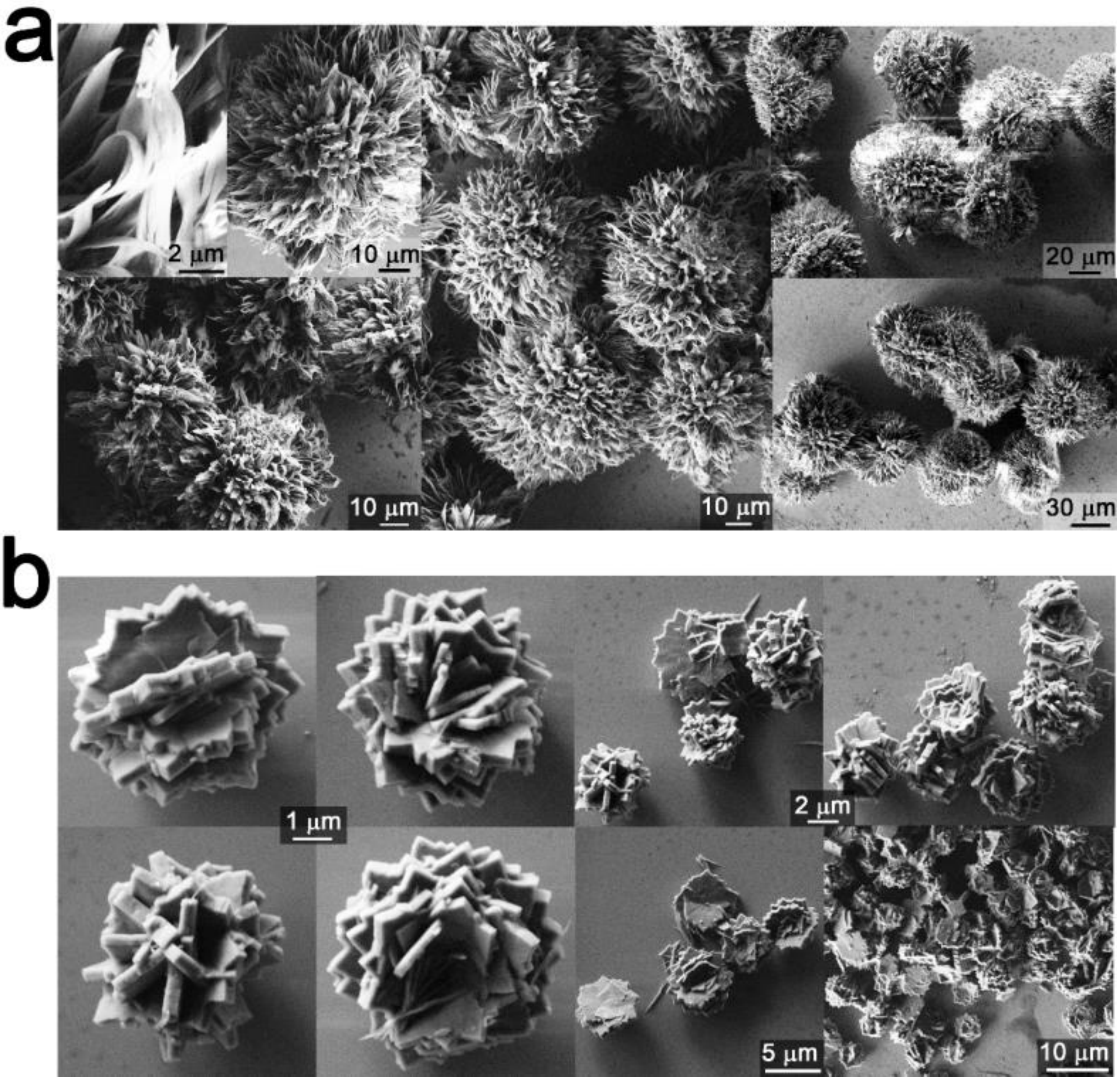

Supplementary Figure 11 | β–QNC hedgehogs. Quinacridone superstructures which were grown into β polymorphic crystal phase.

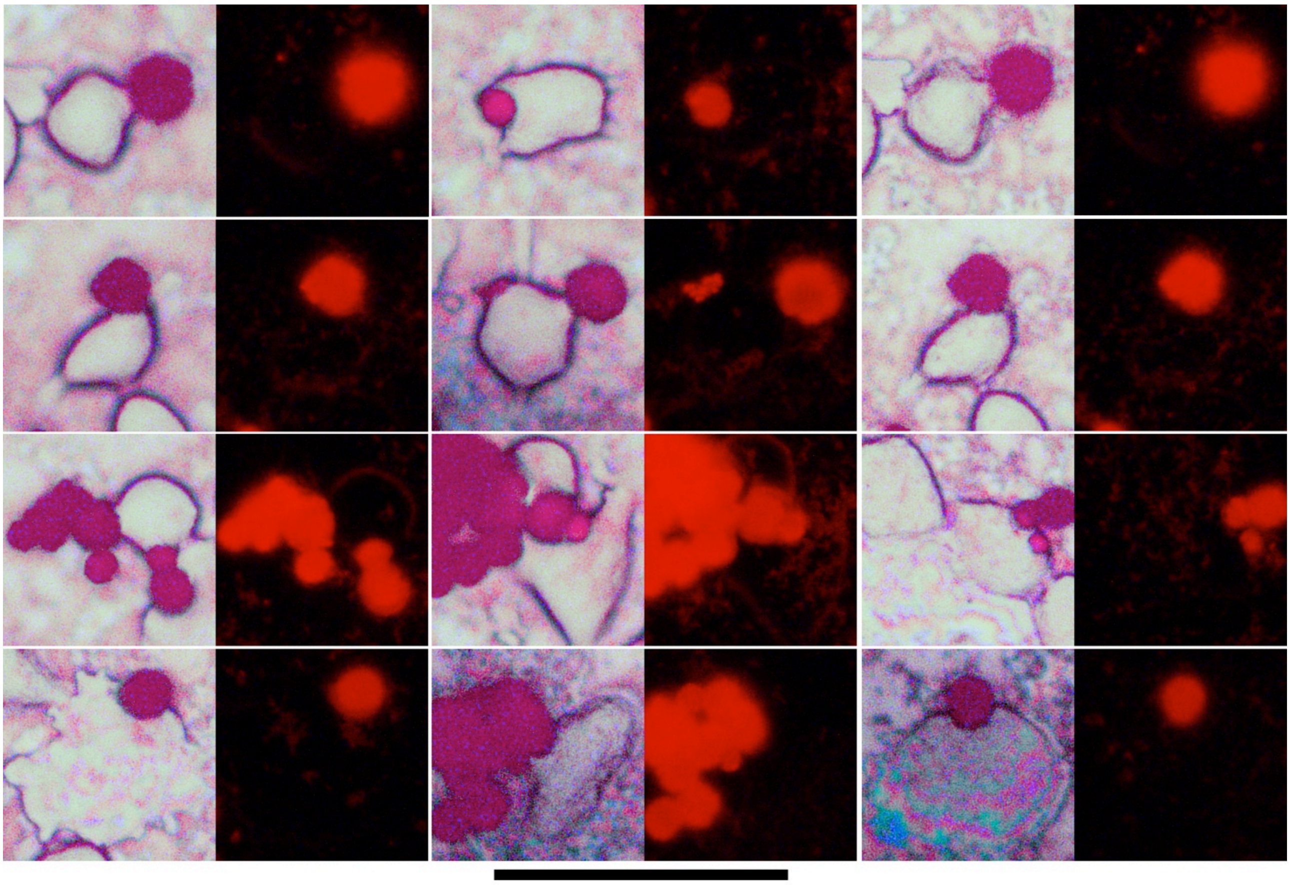

Supplementary Figure 12 | Optical microscopy of RBL cells on hedgehogs, with bright-field images paired with corresponding fluorescence images, showing the luminescence of γ–QNC. Scale bar = 50 μm.

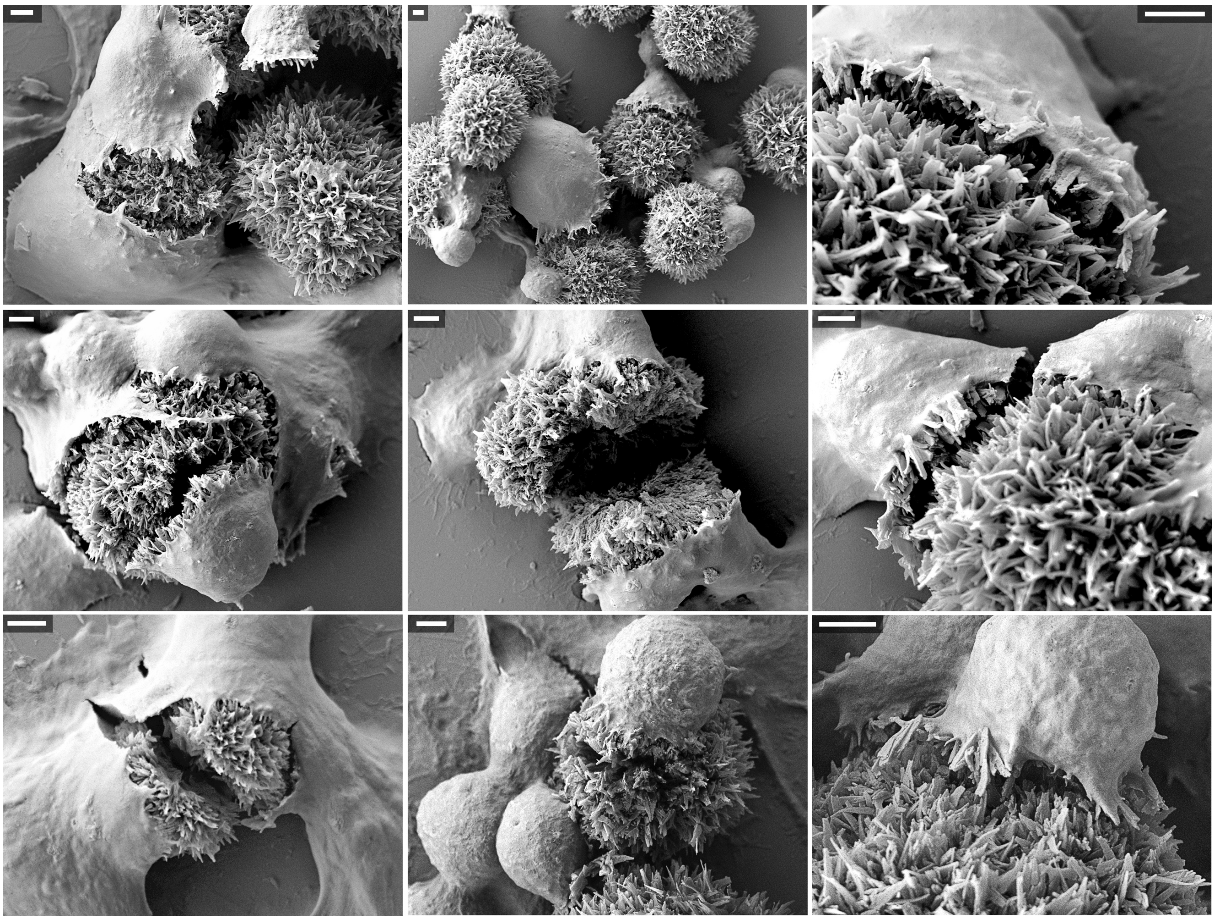

Supplementary Figure 13 | RBL cells deconstructing hedgehogs and rearranging nanocrystallites, shows that though the nanocrystals themselves are rigid, the hierarchical arrangement is plastic. Scale bars = 2 μm.

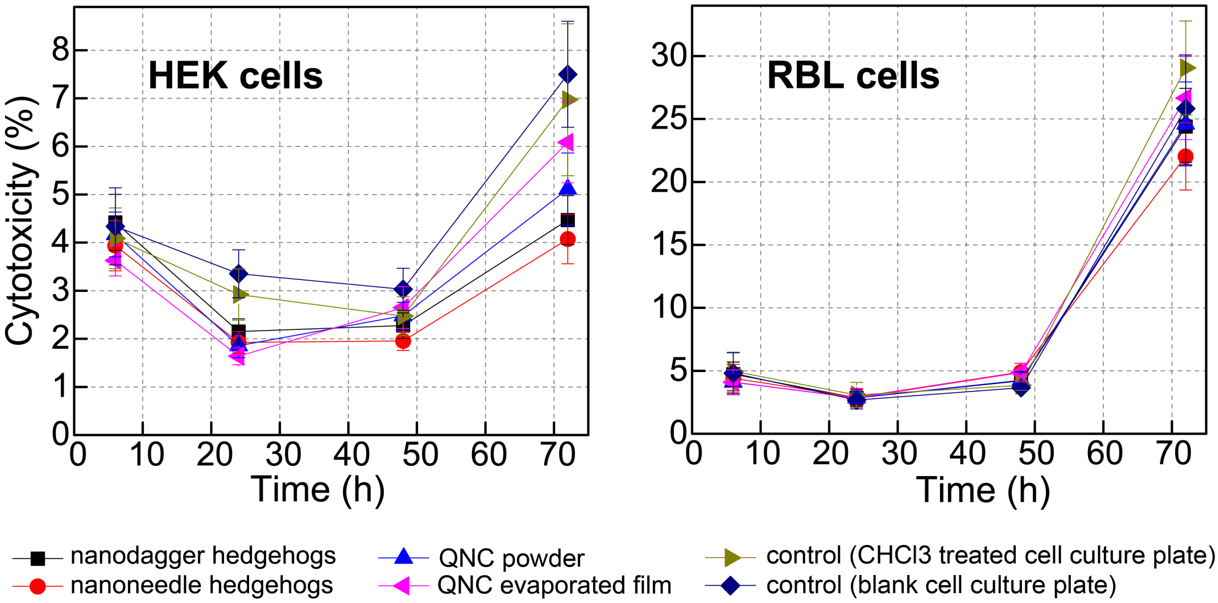

Supplementary Figure 14 | The viability of HEK and RBL cells is not adversely affected by QNC in the form of evaporated thin films, ground powders, or hedgehogs. The CytoTox-Glo™ luminescence-based assay was used (Promega) to allow the viability assays to be carried out and measured directly in cell-culture plates. The last-day data points show increased cell death,

due to overgrowth of cells and depletion of nutrients from the culture media, which was not exchanged over the course of the experiment. (n = 9, error bars show standard error of the mean) For HEK cells (left), control showed slightly higher cytotoxicity than experimental samples, however none of the differences were statistically significant, $p<0.05$ (one-way ANOVA).

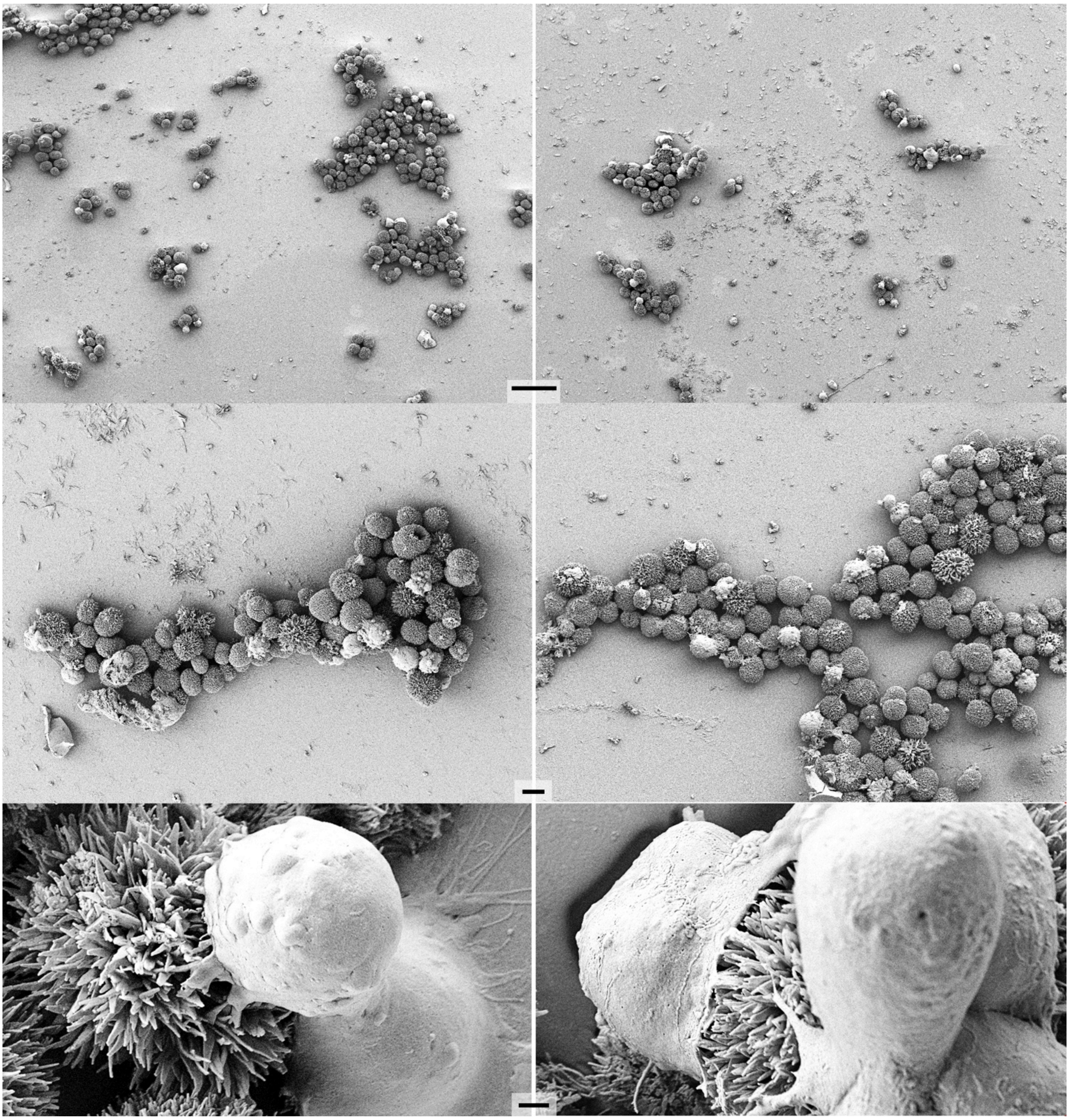

Supplementary Figure 15 | Selective HEK cell growth on hedgehog microstructures. Colloidal solutions of QNC hedgehogs are drop-cast onto a glass slide, after which the entire sample is coated with a uniform vacuum-sublimed QNC layer of 80 nm thickness. Scale bars, from top to bottom rows = 50, 10, 1 μm, respectively.

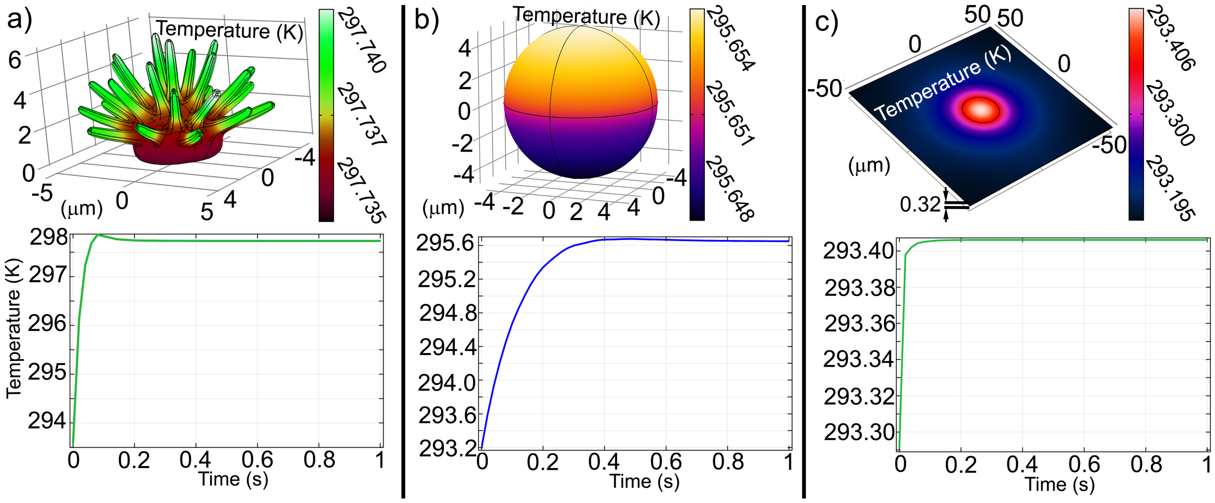

Supplementary Figure 16 | Photothermal heating of different QNC micro-objects. Heating simulations under laser beam were performed with COMSOL Multiphysics 4.2a software (COMSOL Inc.). Heating of different micro objects was modeled by the differential equation for heat transfer in solids by assuming that the radiation energy was absorbed by the surface. The laser power was set as CW 20 mW/cm^2 over 1 second. The heat dissipation was modeled with convective heat flux to the water environment with heat transfer coefficient of 40 $W/(m^2K)$. Hedgehog samples heat up to higher temperature faster than either spheres or planar layers.
